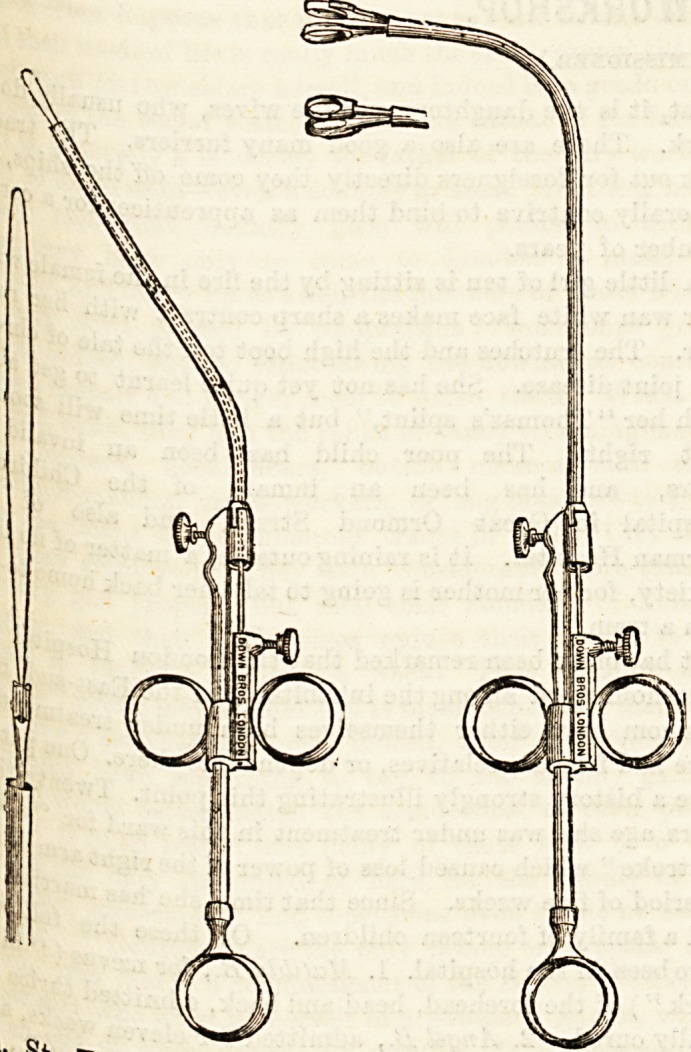# New Drugs, Appliances, and Things Medical

**Published:** 1890-06-21

**Authors:** 


### NEW DRUGS, APPLIANCES, AND THINGS MEDICAL.

[All preparations, appliances, novelties, etc., of which a notice is desired, should be sent to The Editor, The Lodge, Porchester Square, W.]

### MESSRS. DOWN BROTHERS' SURGICAL INSTRUMENTS.

We recently inspected several very ingenious and beautifully made novelties at the above firm, St. Thomas Street, Borough. Amongst them was an idea for the carrying of the various articles of a Nurse's Chatelaine which the nurses will be glad to hear of. All the instruments not having loops naturally have a loop firmly attached to them in the process of manufacture, so that they can be easily detached from the suspending chain, thus doing away with the necessity of carrying a folding case. On the specimen shown to us we noted a probe-thermometer in case, dissecting forceps, tongue depressor, besides scissors and ordinary dressing or polypus forceps, &c.

We also saw a most ingenious nasal snare and laryngeal curette, both working either separately or interchangeably on the same stock. The snare (of which illustrations are given in the next column) is the invention of Professor Krause, of Berlin, and has been introduced into England by Mr. Charters Symonds, of Guy's. The great advantages of the snare are its strength, simplicity, and easy method of being kept clean. The novel way of attaching the wires to a stilet which works in the canula of the instrument is a great improvement on the old plan of bringing the wires separately down the canula to the cross-bar of the thumb-piece. In the latter method the wire, or one side of it, often hung up at the mouth of the canula, necessitating much re-adjustment and consequently loss of time. Here the stilet is firmly screwed to the bar of the ring piece. There is only one part (the stilet) moving in the canula, and that part is firm and moves easily without entanglement.

The laryngeal forceps combine many useful qualities. They are bevelled ring cutting forceps working from a canula, and having antero-posterior and lateral movements. We tested their strength and cutting powers, and found them to fully answer the requirements of the laryngeal surgeon. Messrs. Down also manufacture a useful form of self-retaining soft palate retractor, which is easily adjusted and not irksome to the patient. Amongst the many "stock" instruments which all classes of the profession use we noticed a particularly neat and well-made subcutaneous syringe and case. The syringe is composed of a piece of solid glass tube with the minim divisions clearly marked on it and mounted with well-made fittings, the piston-rod also being graduated. The needles are solid drawn, a great advantage, which Messrs. Down, being manufacturers and not sellers only, are enabled to give the profession. Many hollowed needles are made with seams, and these in time give way, causing the needle to either break easily or to leak. Besides the ordinary hypodemic needles, there is one for exploring to be used with the syringe. This is one of the most useful instruments a doctor possesses, and by it doubtful points of diagnosis are often cleared up. The case is arranged to contain from two to 20 bottles. These are specially made so that the last drop of the solution may be utilized. Huggett's permanent solutions are recommended for them.

## Figures and Tables

**Figure f1:**